# Diabetes, life course and childhood socioeconomic conditions: *an empirical assessment for Mexico*

**DOI:** 10.1186/s12889-024-18767-5

**Published:** 2024-05-09

**Authors:** Marina Gonzalez-Samano, Hector J. Villarreal

**Affiliations:** Tecnologico de Monterrey, School of Government and Public Transformation, EGyTP, Mexico City, Mexico

**Keywords:** Aging, Epidemiological transition, Diabetes, Life course, Childhood conditions, Social determinants of health

## Abstract

**Background:**

Demographic and epidemiological dynamics characterized by lower fertility rates and longer life expectancy, as well as higher prevalence of non-communicable diseases such as diabetes, represent important challenges for policy makers around the World. We investigate the risk factors that influence the diagnosis of diabetes in the Mexican population aged 50 years and over, including childhood poverty.

**Results:**

This work employs a probabilistic regression model with information from the Mexican Health and Aging Study (MHAS) of 2012 and 2018. Our results are consistent with the existing literature and should raise strong concerns. The findings suggest that risk factors that favor the diagnosis of diabetes in adulthood are: age, family antecedents of diabetes, obesity, and socioeconomic conditions during both adulthood and childhood.

**Conclusions:**

Poverty conditions before the age 10, with inter-temporal poverty implications, are associated with a higher probability of being diagnosed with diabetes when older and pose extraordinary policy challenges.

## Background

One of the major public health concerns worldwide is the negative consequences that the demographic (with its epidemiological) transition could bring. This demographic transition is driven by increasing levels of life expectancy (caused by technological innovation and scientific breakthroughs in many cases) and decreasing fertility rates. While during the 20th century, the main health concerns were related to infectious and parasitic diseases, at the present time, non-communicable diseases (NCDs), such as diabetes, constitute a harsh burden in terms of economic and social impact. NCDs most commonly affect the health of adults and the elderly. The economic and social costs associated with NCDs increase sharply with age. These patterns have implications for economic growth, poverty-reduction efforts and social welfare [1].

Mexico’s demographic trends are reflecting a significant shift over the past decades, much like those observed globally. In 1950, the fertility rate stood at 6.7 children per woman, and the proportion of the population aged 60 or over was about 2%. Since the 1970s, there has been a considerable decrease in fertility rates; by 2017, it had dropped to 2.2 children per woman [2]. Even more pressing, according to CONAPO Mexico had a total fertility rate of 1.91 during 2023 [3]. Alongside the declining fertility, the aging population is becoming a more prominent feature in Mexico’s demographic profile. In 2017, individuals aged 60 and over constituted around 10% of the population. Forecasts for 2050 project that this figure will more than double, with those 60 and over representing 25% of the total population. These trends suggest substantial changes in Mexico’s population structure, with implications for policy-making in areas such as healthcare, pensions, and workforce development [2].

Regarding NCDs, in 2017 13% of the Mexican adult population suffered from diabetes, which is twice the Organisation for Economic Cooperation and Development (OECD) average and it is also the highest rate among its members. Some of the risk factors associated with this disease are being overweight or obese, unhealthy diets and sedentary lifestyles. In 2017 72.5% of the Mexican population was overweight or obese [4] and the country had the highest OECD rate of hospital admissions for diabetes. During the period of 2012 to 2017, the number of hospital admissions for amputations related to this condition, increased by more than 10%, which suggests a deterioration in quality and control of diabetes treatments [4]. Moreover, it is estimated that diabetes prevalence will continue with its upward trend; forecasts anticipate that in 2030 there will be around 17.2 million people in Mexico with this condition [5].

Despite the increasing proportion of older people, most of the research regarding the effects of socioeconomic conditions on health focuses on economically active populations. Those which do consider older people, do not investigate length factors such as childhood conditions [6, 7]. In this sense, the Social Determinants of Health (SDH) throughout the Life Course approach provide a framework to ponder and direct the design of public policies on population aging and health [8, 9]. They focus on well-being and the quality of life of populations from a multi-factorial perspective [10–12].

In this study, we explore the impact of childhood and adulthood conditions and other demographic and health aspects on diabetes among older people. The literature has proposed several mechanisms through which the mentioned drivers could operate. In general, these approaches imply that satisfactory socioeconomic outcomes for adults may relatively atone for poor socioeconomic conditions in early childhood [13–16].

Poverty conditions during the first years of life have critical implications, and yet children are twice as likely to live in poverty as adults [16, 17]. On the other hand, poverty is known to be closely linked to NCDs such as diabetes. According to [13], NCDs are expected to obstruct poverty reduction efforts in low and middle-income countries (LMICs) by increasing costs associated with health care. Moreover, the costs resulting from NCDs such as diabetes could deplete household incomes rapidly and impulse millions of people into poverty [16].

The United Nations Children’s Fund (UNICEF) has highlighted the consequences of what it describes as the “invisible epidemic”: non-communicable diseases. NCDs are the leading cause of death worldwide, accounting for 71% or 41 million of the annual deaths globally. The majority (85%) of NCD deaths among people under 70 years of age occur in low and middle-income countries [17].

According the World Health Organization (WHO), SDH are non-medical factors that influence health outcomes, such as the circumstances in which people are born, grow, work, live, and age, and the broader set of forces and systems that shape the conditions of daily life^1^.

These forces include economic policies and systems, development agendas, social norms and policies, and political systems [11, 18]. In this regard, SDH have an important influence on health inequities in countries of all income levels. Health and disease follow a social gradient, that is, the lower the socioeconomic status, a lesser health is expected [11, 18].

On the other hand, the Life Course perspective distinguishes the opportunity to inhibit and control illnesses at key phases of life from preconception to pregnancy, infancy, childhood, adolescence, and through adulthood. This does not follow the health model where an individual is healthy until disease occurs, the trajectory is determined earlier in life. Evidence suggests that age related mortality and morbidity can be anticipated in early life with factors such as maternal diet [19] and body composition, low childhood intelligence, and negative childhood experiences acting as antecedents of late-life diseases [13].

The consequential diversity in the capacities and health needs of older people is not accidental. They are rooted in events throughout the life course and SDH that can often be modified, hence opening intervention opportunities. This framework is central in the proposed “Healthy Aging”. According to WHO [20], Healthy Aging is “the process of developing and maintaining the functional ability that enables well-being in older age”.

In this way, the Life Course and SDH approaches allow to better distinguish how social differences in health are perpetuated and propagated, and how they can be diminished or assuaged through generations. Several research efforts suggest that age related mortality and morbidity can be predicted in early life with aspects such as maternal nutrition, low childhood intelligence, difficult childhood experiences acting as antecedents of late-life diseases [13]. The Life Course acknowledges the contribution of earlier life conditions on adult health outcomes [15, 21]. In addition, SDH have an important influence on inequality and, therefore, on people’s well-being and quality of life [22]. Trends in health literacy across life are also influenced by various SDH such as income, educational level, gender and ethnicity [23].

Finally, though the research that links early life conditions and health outcomes in adulthood is scarce in low and middle-income countries, our study aims to address the gaps in knowledge regarding the impact of childhood socioeconomic conditions on long-term health outcomes, including the prevalence of non-communicable diseases in LMICs. We specifically focus on the incidence of diabetes in Mexico. Advocating for early-life targeted interventions, we highlight the critical need to address the root causes of NCDs to reduce their impact on the most vulnerable groups. Utilizing data from the Mexican Health and Aging Study (MHAS), which provides comprehensive health, demographic, and socioeconomic information on individuals aged 50 and older, as well as details on their childhoods (before the age of 10) and family health backgrounds [24], our research emphasizes the importance of developing targeted interventions on early life course stages.

### Health, childhood and adulthood conditions

Multiple studies highlight that childhood experiences can influence patterns of disease, aging, and mortality later in life [10, 11, 16, 20, 25]. The conditions in health and its social determinants accumulate over the life course. This process initiates with pregnancy and early childhood, continues throughout school years and the transition to working life and later in retirement. The main priority should be for countries to ensure a good start in life during childhood. This requires at least adequate social and health protection for women, plus affordable good early childhood education and care systems for infants [11].

However, demonstrating links between childhood health conditions and adult development and health is complex. Frequently, researchers do not have the data necessary to distinguish the health effects of changes in living standards or environmental conditions with respect to childhood illnesses [26]. A study conducted in Sweden, concluded that reduced early exposure to diseases is related to increases in life expectancy. Additionally, research with data from two surveys of Latin America countries found associations between early life conditions and disabilities later in life. In this sense, the study suggests that older people who were born and raised in times of poor nutrition and a higher risk of exposure to infectious diseases, were more likely to have some disability. In a survey in Puerto Rico, it was observed that the probability of being disabled was greater than 64% for people who grew up in poor conditions than for those who grew up in good conditions. Another survey that considered seven urban centers in Latin America found that the probability of disability was 43% higher for those with disadvantaged backgrounds, than for those with favorable ones [26].

Recent studies have focused on childhood circumstances to explain later life outcomes [12, 27–31]. These research findings have shown the importance of considering socioeconomic aspects during childhood, including child poverty from a multidimensional perspective [12], as a determinant of health status of adults and health disparities. When disadvantaged as children, irreversible effects on health show-up frequently. One clear example is the association of socioeconomic aspects during childhood with type 2 diabetes and obesity in adulthood [32, 33].

The future development of children is linked to present socioeconomic levels and social mobility in adulthood [27]. Some studies [28, 34, 35] indicate that the effects of childhood exposure to lower socioeconomic status or conditions of poverty on health in old age may persist independently of upward social mobility in adulthood. Hence, children who grow up in poverty are more likely to present health problems during adulthood, while those who did not grow up in poverty have a higher probability of remaining healthy.

Another important consideration regards developmental mismatches [36]. Their article emphasizes how developmental and evolutionary mismatches impact the risk of diseases like diabetes. There could be a disparity between the early life environment and the one encountered in adulthood, turning adaptations that were once beneficial into risk factors for non-communicable diseases. High-calorie diets and sedentary lifestyles could trigger diabetes prevalence.

If these connections between early life and health in old age can be established firmly, it is expected that aging people in low and middle-income countries have another disadvantage regarding elders in developed countries, including a higher risk of developing health problems in old age and frequently multiple NCDs [26]. Under this context, the effective management of NCDs such as diabetes is crucial, and childhood living standards would be a variable to ponder [26, 37]. Work related to the Life Course approach has emphasized the importance of considering socioeconomic aspects during childhood, including poverty [12] as a determinant of adult health status and its disparities [28–31].

## Data and methods

### Data source

The Mexican Health and Aging Study (MHAS) is a national longitudinal survey of adults aged 50 years and over in Mexico. The baseline survey has national, urban, and rural representation of adults born in 1951 or earlier. It was conducted in 2001 with follow-up interviews in 2003, 2012, 2015, 2018 and 2021 [38]. New samples of adults were added in 2012 and 2018 to refresh the panel. The survey includes information on health measures (self-reports of conditions and functional status), background (education and childhood living conditions), family demographics, and economic measures. The MHAS (Mexican Health and Aging Study) is partly sponsored by the National Institutes of Health/National Institute on Aging (grant number NIH R01AG018016) in the United States and the Instituto Nacional de Estadística y Geografía (INEGI) in Mexico. Data files and documentation are public use and available at www.MHASweb.org.

In this research, the analysis was based on data from the survey conducted in 2018 (it was the most recent when the project started, later the 2021 survey became available). The study focused exclusively on participants who were aged 50 or older at the time of the 2018 survey. To minimize response bias, the study included only observations from direct interviewees, excluding proxy respondents, and particularly those who completed the section of the questionnaire pertaining to “Childhood Characteristics before the age of 10 years”^2^. Furthermore, to expand the sample size, individuals who first joined the survey during the 2012 cycle were identified, utilizing data from both the 2012 and 2018 surveys [39]. After locating the same individuals in both datasets, responses related to childhood conditions from the 2012 survey were extracted and integrated into the 2018 dataset. Biases in the samples were not found. This approach resulted in a total sample size of 8,082 observations.

In addition, we selected a suite of predictor variables to provide a comprehensive examination of the demographic, socioeconomic, and health-related characteristics within our sample (Table 1). The cohort consists of 8,082 participants with males exhibiting a marginally higher mean age (58.3 years) compared to females (56.7 years). In terms of educational achievement, males attained a slightly higher level of schooling, averaging 8.3 years, as opposed to 7.6 years for females.

**Table 1:** Characteristics of the studied sample by gender

Variable	Females (*N*=4,368)	Males (*N*=3,714)
**Demographic and Socioeconomic**		
Age (mean)	56.7	58.3
Years of school (mean)	7.6	8.3
Urban locality^a^ (%)	80.3	77
Rural locality^a^ (%)	19.7	23
Living with a couple (%)	68.8	86
Single^b^ (%)	31.2	14
Proxy: No poverty in adulthood (%)	51.2	50.1
Proxy: Childhood poverty (%)	62.4	63
Diabetes diagnosed (%)	24.4	20.1
Use of insulin to control diabetes (%)	26.6	22.1
**Health**		
Use of medicine to control diabetes (%)	91.5	85.3
Condition of obesity (Body Mass Index>= 30) (%)	34.8	24.6
Mother with diabetes (%)	32.6	31.5
Father with diabetes (%)	20	19

^a ^According to the National Institute of Statistics and Geography (INEGI), a population is considered rural when it has fewer than 2,500 inhabitants, while urban is where more than 2,500 people live. Retrieved from http://cuentame.inegi.org.mx/poblacion/rur_urb.aspx?tema=P. Accessed on January 20, 2024

^b^ Single refers to not living with a partner at the time of the interview, although the person could have been married, divorced, separated, or never in a cohabiting relationship

Regarding the spatial distribution of the study population reveals that 1,717 individuals reside in areas with 2,500 inhabitants or fewer, indicating a rural setting, while the majority, 6,365 individuals, are found in regions with more than 2,500 inhabitants, suggesting an urban setting. Among the subjects, a significant number of males (23%) are located in the former, rural settings, which is higher than their female counterparts (19.7%). The data on living arrangements indicate notable gender differences, with 86% of males cohabiting with partners against 68.8% of females. The state of being single-a term here encompassing a spectrum of prior marital experiences but currently not cohabiting-is observed in 31.2% of females and 14% of males. The socioeconomic dimension is gauged using “proxy variables” such as the absence of poverty in adulthood and presence of childhood poverty, both of which are evenly represented across genders. Health-related self-reporting data reveals that females have a higher incidence of diagnosed diabetes (24.4%) compared to males (20.1%), and a larger percentage of females (26.6%) manage their diabetes with insulin. The propensity for medication use to control diabetes is high among both sexes, though more pronounced in females (91.5%) relative to males (85.3%). Additionally, obesity rates, determined by a Body Mass Index^3^ of 30 or greater, are substantially elevated in females (34.8%) versus males (24.6%). Furthermore, a familial history of diabetes is slightly more prevalent in females, affecting 32.6% with diabetic mothers and 20% with diabetic fathers.

There is a serious concern about self-reporting medical conditions, to what extent this information is reliable. For [40, 41] the validity and high accuracy of self-reported diagnosis of diabetes mellitus has been confirmed by previous research, and previous studies using WHO data have also used this question to evaluate diabetes mellitus [42, 43].

For the survey employed in this paper, [44] confirm a correspondence between self-reported and objective measures. Nonetheless, [45] warn about true prevalence and this kind of reporting. In addition, the implications of relying on diagnosed diabetes, rather than total diabetes prevalence, include the potential under-representation of the condition’s true prevalence due to undiagnosed cases. Since the study’s analysis is based on self-reported data from the Mexican Health and Aging Study, it might not capture those individuals who are unaware of their condition [45]. The existence of statistical biases could be a potential limitation in the analysis.

Equally or even more troublesome is the problem of recalling conditions during childhood. While some factors (depression among others) can produce limited recalling [46], specific conditions are well recalled, if not their details and timing [47].

Regarding the age distribution, the sample is mostly concentrated in three groups: 67.6% for individuals between 50 and 59 years of age, followed by 29.6% for those between 60 and 69 years of age, and 2.5% for those between 70 and 79 years of age. On average, the educational level for women is 7.6 years of schooling while for men it is 8.2 years, which suggests an incomplete level of secondary education for both. On the other hand, from the total number of women in the sample (4,368), 24% of them indicated the presence of diabetes, and 20% of men in the sample (3,714) reported this condition. In addition, around 68% of women with diabetes reported being overweight or obese, for men this percentage was 69%. Meanwhile, 71.4% women with diabetes reported parental history of diabetes, for men this percentage was 68%. The next subsections describe the construction and identification of the key dependent and independent variables.

#### Dependent variable

The dependent variable is binary, which refers to the individual’s diagnosis of diabetes. This variable was taken from section C of the basic questionnaire of the MHAS 2018. The question is as follows: Has a doctor or medical professional ever told you that you have diabetes? If the answer is “yes” it was assigned a value of 1 and if the answer was “no”, a 0. The absence of answers was left empty, non-imputed. Regarding the individuals who reported being diagnosed with diabetes, 94.2% were taking medication or using insulin injections or pumps, and / or following a special diet to manage diabetes, without statistical differences when interchanging the samples.

#### Independent variables

For the explanatory variables of the model, sociodemographic, socioeconomic (“proxy”^4^ of poverty in childhood and non-poverty in old age)^5^, and geographical variables were considered, as well as other variables related the parents of the interviewees. Given the difficulty of constructing a robust variable that reflects respondents’ income, internet access was considered as a proxy variable that would allow to ascertain the poverty status of the individual in old age. Several tests were performed for robustness^6^.

Internet access in Mexico is more common among relative well-off Mexicans than it was among the poorest sector of the population. Thus, according to [49, 50], 7 out of 10 individuals from the highest income segment were internet users, while for the lowest income deciles, this was only 2 out of 10. Furthermore, a low level of schooling was related to internet access opportunities. Therefore, people who only received primary education were 4 times less likely to use the internet in Mexico.

Additionally, for the variable of poverty during childhood, a proxy was considered which corresponds to the answer of the question “Before you were 10 years old, did your home have an indoor toilet?”^7^, United Nations Children’s Fund (UNICEF) collaborators [12], pointed out that the severe deprivation of sanitation facilities has critical long-term effects on various aspects of an infant. In this regard, UNICEF highlights the crucial importance of eradicating severe sanitation deprivation as a method to eradicate absolute child poverty, emphasizing that sanitation facilities should be a priority for children.

### Statistical analysis

Linear Probability Models (LPM) define the probability:

1 $$\begin{aligned} Pr(Y=1 \mid X)=\beta _{0}+\beta _{1}X_{1} \end{aligned}$$

They assume (require) that: i) $Pr(Y=1 \mid X)$ is an increasing function in *X* for $\beta _{0}>0$, and ii) $0 \le Pr(Y=1 \mid X) \le 1 \forall X$.

This implies a cumulative distribution function that guarantees that for any value of the parameters of *X*, probabilities are well-defined, with values in the interval [0, 1].

The dependent variable to be explained is binary (diabetes diagnosis is 1 if the person has been diagnosed with diabetes and 0 for the person who has not been diagnosed with diabetes). Hence, a special class of regression models (with limited dependent variable), is considered. There are two probability models with these characteristics frequently used: the Logit model, and the Probit model. In relation to this, [48] points out that, theoretically, both models are very similar. A potential advantage of Probit models is they could feed other related inquiries. For example, when testing selection via Inverse Mill’s Ratios.

The Probit model is expressed as:

2 $$\begin{aligned} Y=\beta _{0}+\beta _{1}X_{1}+\beta _{2}X_{2}+...+\beta _{k}X_{k} \end{aligned}$$

In the Probit model with multiple regressors, $X_1,X_2,\ldots ,X_k$, $\phi (.)$ the cumulative standard normal distribution function is $\phi (Z)=P(X\le z)$ , $Z\sim N(0,1)$.

Therefore, in (2) $P(Y=1 \mid X_1,X_2,\ldots ,X_k )$ means the probability that an event occurs given the values of other explanatory variables, where Z is distributed as a standard normal $Z\sim N(0,1)$. While a series of tests could be performed in the model, two are critical for this investigation: the linearity between the independent variables and the underlying latent variable, and the normality of errors.

In (2), the coefficient $\beta _{1}$ represents the change in *z* associated to a unit of change in $X_1$. It is then observed that, although the effect of *z* on a change is linear, the link between *z* and the dependent variable *Y* is not linear since $\phi$ is a non-linear function of *X*. Therefore, the coefficients of *X* do not have a simple interpretation. In that sense, marginal effects must be calculated. Considering that in the linear regression model, the slope coefficient measures the change in the average value of the returned variable, due to a unit of change in the value of the regressor, maintaining the other variables constant. In these models, the slope coefficient directly measures the change in the probability of an event occurring, as a result of a unit change in the value of the regressor, holding all other variables constant, a discussion can be found at [51]. The $\beta$ parameters are frequently estimated by maximum likelihood. The likelihood function is the joint probability distribution of the data treated as a function of the unknown coefficients^8^.

The maximum likelihood function is the conditional density of $Y_1,\ldots ,Y_k$ given $X_1,\ldots ,X_k$ as a function of the unknown parameters $\beta$. Thus, the Maximum Likelihood Estimation (MLE) is the value of the parameters $\beta$ that maximizes the maximum likelihood function. Hence, the MLE is the value of $\beta$ that best describes the distribution of the data. In this regard and in large samples, the MLE is consistent, normally distributed, and efficient (it has the lowest variance among all the estimators). The $\beta$ is solved by numerical methods. The resulting ${\hat{\beta }}$ is consistent, normally distributed, and asymptotically efficient.

### Model

A Probit model is proposed as follows. The dependent variable is diagnosed diabetes in adulthood correlated to several independent variables: sex, age, marital status, locality size, a dummy variable (to identify observations sourced from the 2012 survey wave, which is focused on childhood-related questions), obesity condition (Body Mass Index $\ge$ 30), family history of diabetes, childhood poverty, no poverty in adulthood and the interaction of childhood poverty and no poverty in adulthood.

$$\begin{aligned} P(diabetes\ diagnosed \mid x) = \phi (sex,\ age,\ marital\ status,\ locality\ size,&\\ tipent\_12\_dummy,\ obesity,\ family\ history\ of\ diabetes,&\\ chilhood\ poverty,\ no\ poverty\ in\ adulthood,&\\ chilhood\ poverty*no\ poverty\ in\ adulthood)&\end{aligned}$$

The variables should have analogous probability distributions and behave mutually independent. If errors violate the assumptions, the estimated values would be biased and inconsistent. Therefore, estimated values will also be shown with the Linear Probability Model.

In this type of model, $y_i$ is a latent dependent variable that takes values of 1 if the person has been diagnosed with diabetes, that is, if individual *i* has a certain characteristic or quality and 0 otherwise; *X* is a set of explanatory variables that are assumed to be strictly exogenous, which implies that $Cov\left[ x_i,\varepsilon _j\right] =0\ \forall$ the *i* individuals. In addition, the error term $\varepsilon$ is assumed to be *i*.*i*.*d*. In this way, the probability of an event occurring given a set of explanatory variables is obtained:

$$\begin{aligned} P(y_{it}=1 \mid x_{it}){} & {} = G\left( \beta _0 + \beta _1 X_{1i,t} + \beta _2 X_{2i,t} + \beta _3 X_{3i,t} *\beta _4 X_{4i,t} + \ldots \right. \\{} & {} \quad \left. + \, \beta _k X_{ki,t} + \varepsilon _{it} > 0\right) \end{aligned}$$

In (1) *G* is a function that strictly takes values between 0 and 1, $0<G(z)<1$, for all real numbers *z*. As noted at the beginning of this section, in the Probit model, *G* represents a standardized normal cumulative distribution function given by:

$$\begin{aligned} G(Z) = \int _{-\infty }^{z} \frac{1}{\sqrt{2\pi }} \exp \left( \frac{-t^{2}}{2}\right) dt \end{aligned}$$

Finally, to know the effects of the changes in the explanatory variables on the probability of the event occurring, a partial derivative can show that:

$$\begin{aligned} \frac{\partial p\left( y=1\mid x\right) }{\partial x_j}=g(\beta _0+x\beta {)\beta }_j,\ where\ g\left( z\right) =\ \frac{\partial G}{\partial z}\ (z) \end{aligned}$$

The term $g\left( z\right)$ corresponds to a probability density function. Since the Probit model $G\left( .\right)$ is a strictly positive cumulative distribution function, $g\left( z\right) >0\ \forall \ z$, the sign of the partial effect is the same as that of $\beta _j$.

## Results

This section reviews the factors associated with the probability of being diagnosed with diabetes for men and women and discusses their significance. Table 2 summarizes the main results of the Probit model.

**Table 2:** Probit model results

Variables	Coefficients	Marginal effects
Proxy: childhood poverty^a^	0.09	0.02
	(0.06)	(0.02)
Proxy: no poverty in adulthood^a^	-0.18 ***	-0.05 ***
	(0.06)	(0.02)
Sex: Woman	0.16 ***	0.04 ***
	(0.03)	(0.01)
tipent_12_dummy^b^	0.10 **	0.03 **
	(0.05)	(0.01)
Living with a couple	0.12 **	0.03 **
	(0.04)	(0.01)
Urban locality (with 2,500 inhabitants or more)	0.18 ***	0.05 ***
	(0.05)	(0.01)
Age	0.02 ***	0.01 ***
	(0.00)	(0.00)
Mother with diabetes	0.43 ***	0.13 ***
	(0.04	(0.01)
Father with diabetes	0.40 ***	0.12 ***
	(0.04)	(0.01)
Condition of obesity (Body Mass Index $\ge$ 30)	0.14 ***	0.04 ***
	(0.04)	(0.01)
Interaction:childhood poverty*no poverty in adulthood	0.15 **	0.05 **
	(0.08)	(0.02)
**N**	6,746	

^a^ The term “proxy”, was employed to describe variables that serve as stand-ins for factors that are not directly observable within our dataset. As noted by [48]

^b ^This is a binary indicator used in our dataset to distinguish observations collected during the 2012 survey wave, particularly those pertaining to questions related to childhood conditions

Significance levels: $$*** p<0.01, ** p<0.05, * p <0.10$$. Robust standard errors are reported in parentheses. Marginal effects represent the partial effects for the average observation

### Sociodemographic

Marginal effects on the dependent variable show that the age of individuals is highly significant with a positive correlation. This suggests that age is a factor leading to a higher probability (1%) of obtaining a diagnosis of diabetes, which could imply that as the person ages, the likelihood of developing diabetes increases. This result is consistent with studies conducted on the age-related decline in mitochondrial function, which in turn contributes to insulin resistance in old age. These conditions may foster the development of glucose intolerance and type 2 diabetes [53, 54].

In addition, the outcomes indicate that women have an associated probability increase of 4% of suffering from this disease compared to men^9^. Regarding the differences by marital status, women and men living in a couple have a higher probability of being diagnosed with diabetes. In a study for Mexico using MHAS 2012, [45] found that being a woman and being married are significantly associated with a higher likelihood of self-reported diabetes^10^.

On the other hand, the results by size of locality suggest that individuals residing in urban areas have a non-negligible higher probability of suffering from diabetes compared to people living in rural locations. This is in line with the phenomenon of “nutritional transition”, which initially occurred in high-income countries and later in low-income countries, first in urban areas and then in rural areas [56, 57]. For Mexico, [58] despite the prevalence of diabetes presents heterogeneous patterns, this condition is strongly greater in urban areas compared with rural areas.

### Health and lifestyle

The results suggest a significant positive effect on the probability of diagnosis of diabetes for the individuals in the sample when the father and/or mother have this condition. In the case of a mother with diabetes, the associated probability of diabetes is 13%, while for a father with diabetes, it is 12%. Additionally, obesity is an important risk factor in the diagnosis of diabetes, the linked marginal effect of this comorbidity in the diagnosis of diabetes is 4%. In this regard, no significant differences were found by sex or locality size^11^.

### Socioeconomic

The findings indicate a lower probability that individuals are diagnosed with diabetes if during adulthood they are not poor (-5%). On the other hand, from the interaction of the variables poverty in childhood and non-poverty in old age, a considerable positive effect is observed. This suggests that when the individual was poor in childhood, despite no longer poor in adulthood, the probability associated with the diagnosis of diabetes is positive and significant. Thus, it is possible that conditions of poverty in childhood influence the development of this disease later in life^12^. While this is a correlation, the fact that an interaction of socioeconomic characteristics has bigger linear effect than a key biological characteristic (obesity) is non trivial, and reinforces the importance of life course analysis.

Social mobility, defined as the change in an individual’s socioeconomic status relative to their parents or over their lifetime, is a crucial metric for assessing equal opportunity-a measure of whether people have the same chances to achieve success regardless of their initial socioeconomic position. Our study aligns with the broader evidence [65, 66], suggesting that those from disadvantaged backgrounds often face significant barriers to socioeconomic advancement^13^.

## Discussion

A compelling finding of this paper, refers how poverty conditions during childhood remain an important risk factor associated with the greater probability of being diagnosed with diabetes during adulthood in Mexico. Despite these circumstances do not determine the diagnosis of diabetes in older adults, they have a strong correlation with the ailment. On the other hand, even when individuals have not experienced poverty during childhood, but it occurs during adulthood, the probability associated with the diagnosis of diabetes increases. Not surprisingly, the probability of being diagnosed with diabetes scales when the person was poor in both stages. These effects are persistent for men and women, although for women the associated probability was higher than for men. Likewise, there is a positive and high correlation of the parents’ history of diabetes and the obesity condition on the probability of developing this disease. Biological aspects could be present, but also modifiable factors, with the generational transmission of elements related to lifestyle (eating habits and physical activity). Similarly, people who live with a partner have a higher associated probability of being diagnosed with diabetes. The literature suggests that this is due to the tendency of individuals to select spouses based on the preference for similar phenotype characteristics and the convergence of their behaviors and lifestyle. Moreover, these issues have been exacerbated by urbanization processes and by the “food transition”^14^ that has made processed and ultra-processed products more and more accessible. Such products are characterized by being high in fat, salt, and sugar. Regarding the effect of the size of the locality on the probability of being diagnosed with diabetes, the results show differences for people residing in rural and urban areas. In urban localities, the associated probability is higher compared to rural ones. Likewise, aging is an important factor that affects the probability of suffering from diabetes: as the individual ages, the probability of developing this disease increases.

In terms of the analysis and empirical strategy used, the findings show valuable relationships. Aligned with efforts to improve the accuracy and reliability of health data by combining biomarkers and objective measurements with self-reported data [70], biomarkers in the survey were employed. These biomarkers were used for diabetes (the dependent variable) and obesity condition (as one of the independent variables) in the model of Results section. The results are consistent with the previous findings (See Appendix).

There is ample space for additional work and get over the limitations of this work. For example, being MHAS a longitudinal survey, an econometric model can be developed in order to explore (test) causal relationships among the extensive set of variables. Also self-reporting could present different types of biases. While the use of biomarkers was an important robustness test, calculating bounds and checking selection biases would be valuable. Moreover, the survey also captures information related to social protection variables and social programs transfers, which could be useful for testing policies.

## Conclusions

Given the interconnection of childhood conditions and the importance of these in the development of adult capacities and their success in their future life, they should be considered within the design and formulation of public policies and programs. The policies should focus and prioritize objectives of reducing the inequality gaps and pre-existing poverty in the country. Adopting measures to reduce inequalities in the social sphere is essential to protect future generations. In this sense, it is important to act on the Social Determinants of Health throughout the course of life in a broader social and economic context. Acting on the SDH would improve prospects for health and generate considerable social benefits that would allow people to achieve their capabilities and reduce the intergenerational perpetuation of inequalities. Thus, the SDH together with the Life Course approach, provide a sensible framework to identify risk clusters that can be broken in periods of effective interventions (e.g. childhood), as well as to improve the design of public policies on population aging and health, from a perspective focused on the well-being and quality of life of the Mexican population.

In this way, and to face the demographic transition and the diabetes epidemic in Mexico, comprehensive public policies that consider interventions from childhood will be required to reduce inequality and poverty. For some years now, the WHO has emphasized the importance and role of the inclusion of long-term care policies and programs focused on older adults. The forecasts in case of untimely acting indicate a significant negative effect on the social, economic and health structures for the coming years.

Finally, despite the increase of older population, much of the research on the effects of socioeconomic conditions on health is concentrated in economically active populations, and those ignore older people, and pay restricted attention to long term factors such as childhood conditions. The results presented in this document contribute to studies on population aging and public health. Evidence is found with respect to health determinants in a demographic group that is growing rapidly and not sufficiently considered.

## Data Availability

Data files and documentation are public use and available at www.MHASweb.org. Data and code used during the current study are available from the corresponding author on reasonable request.

## Appendix

For robustness testing a model specification was employed where self-reported diabetes and obesity measures are substituted with biomarkers obtained from the MHAS 2012. Table 3 summarizes the main results of the Probit model.

The analytical results from Table 2 (Model 1), and those derived from the utilization of biomarkers in Table 3 (Model 2) exhibit a considerable likeness, especially in the context of diabetes and obesity indicators. Notably, there is a significant reduction in the sample size when biomarkers^15^ are introduced, which might account for the increased standard errors observed in Table 3. Consequently, certain variables such as: being “woman”, “living with a couple” and “residing in an urban locality”, have lost statistical significance in the biomarker analysis. Despite these differences, the general conclusions derived from this specification remain consistent with those presented in Model 1 (Table 2). Moreover, the linear effect of the interaction effect of poverty in childhood with no poverty in adulthood is bigger with the biomarker specification. Nonetheless, the larger confidence intervals need to be considered.

**Table 3:** Probit Model Results using Biomarkers (MHAS, 2012)

Variables	Coefficients	Marginal effects
Proxy: childhood poverty^a^	0.09	0.03
	(0.18)	(0.06)
Proxy: no poverty in adulthood^a^	-0.33 **	-0.12 **
	(0.19)	(0.07)
Sex: Woman	0.21 **	0.08 **
	(0.11)	(0.04)
Living with a couple	0.12	0.04
	(0.12)	(0.04)
Urban locality (with 2,500 inhabitants or more)	0.14	0.05
	(0.13)	(0.04)
Age	0.02 ***	0.009 ***
	(0.01)	(0.004)
Mother with diabetes	0.32 ***	0.12 ***
	(0.12)	(0.04)
Father with diabetes	0.40 ***	0.15 ***
	(0.14)	(0.05)
Condition of obesity (Body Mass Index $\ge$ 30)	0.49 ***	0.18 ***
	(0.11)	(0.04)
Interaction:childhood poverty*no poverty in adulthood	0.41 **	0.15 **
	(0.23)	(0.09)
**N**	601	

^a^ The term “proxy”, was employed to describe variables that serve as stand-ins for factors that are not directly observable within our dataset. As noted by [48]

**Notes**: Significance levels: $$*** p<0.01, ** p<0.05, * p <0.10$$. Robust standard errors are reported in parentheses. Marginal effects represent the partial effects for the average observation

## Abbreviations

MHAS: Mexican Health and Aging Study
NCDs: non-communicable diseases
SDH: Social Determinants of Health
WHO: World Health Organization
OECD: Organisation for Economic Cooperation and Development
INEGI: National Institute of Statistics and Geography
UNICEF: United Nations Children’s Fund
LMICs: Low and middle-income countries
LPM: Linear Probability Models

## Footnotes

1 Social Determinants of Health. Retrieved from https://www.who.int/health-topics/social-determinants-of-health#tab=tab_1. Accessed on January 22, 2024.

2 Given the survey design, people responding the childhood questionnaire are new participants.

3 A Body Mass Index (BMI) was constructed considering the variables of height and weight reported in the MHAS 2018 survey (C6: “What is your current weight in kilograms?”, C67: “What is your height without shoes in meters?”). For adults, the World Health Organization (WHO) defines overweight as a BMI of 25 or higher, and obesity as a BMI of 30 or higher. BMI was calculated by dividing a person’s weight in kilograms by the square of their height in meters (kilograms/m^2^).This information is available at: https://www.who.int/es/news-room/fact-sheets/detail/obesity-and-overweight, accessed on January 10, 2024.

4 In this context, the term “proxy”, was employed to describe variables that serve as stand-ins for factors that are not directly observable within our dataset. As noted by [48].

5 Numerous variables that could reflect household income were tested, but since they were self-reported and not part of the survey’s core, there is a large number of missing values.

6 We thank one referee for her suggestions regarding education years.

7 This question is found in section J.18 of the basic questionnaire and corresponds to the question “Does this home have ... internet?” If the person answers “yes”, that means that they have internet service and were assigned a value of 1, and 0 if the person does not have this service.

8 There is an interesting possibility of comparing the linear marginal effects with direct estimations from a Logit model (risk differences), [52]. We thank a referee for pointing this out.

9 This is consistent with what was stated in Aging in Mexico: The Most Vulnerable Adults of the MHAS Newsletter: May 20-2, 2020, which indicates that women are more likely to report diabetes than men. Retrieved from http://www.enasem.org/images/ENASEM-20-2-Aging_In_Mexico_AdutosMasVulnerables_2020.pdf. Accessed on February 10, 2024.

10 Furthermore, Danish researchers found a connection between the Body Mass Index of one spouse and the other spouse’s risk of developing type 2 diabetes. According to this study, spouses tend to be similar in terms of body weight, as people often tend to marry someone similar to themselves and share dietary and exercise habits when living together [55].

11 It has long been known that type 2 diabetes is, in part, hereditary. Family studies have revealed that first-degree relatives of people with type 2 diabetes are approximately 3 times more likely to develop the disease than people without a positive family history of the disease [59–61]. Likewise, in a study for Mexico, [62] point out that obesity and a history of type 2 diabetes in parents and genes play an important role in the development of type 2 diabetes. Furthermore, [63], points out that the frequency of diabetes mellitus also varies between different races and ethnicities.

12 This is consistent with the research by [64] who find that the conditions in which the person lived at the age of 10 affect health in old age.

13 According to [67] in a regional analysis on the degree of social mobility in Mexico, it indicates that social mobility is higher than the national average in the North and Central North regions, similar to the national average in the Central region, and lower than the average in the South region. In particular, it notes that children of poor parents made above-average progress if they grew up in the northern region, and less than average progress if they grew up in the southern region.

14 The country’s food environment has been transformed; it is becoming easier to access unhealthy products. In this sense, for the last 40 years, important changes have been observed in the Mexican diet, mainly from fresh and unprocessed foods to processed and ultra-processed products with a high content of sugar, salt, and fat. Marrón-Ponce et al. [68], point out that in 2016 around 23.1% of the energy in the Mexican population’s diet came from ultra-processed products, even though the WHO recommendations suggest that at most, this percentage should present between 5 and 10% of total energy per day. In addition, Mexico is the worldwide largest consumer of sugary beverages; its consumption represents approximately 10% of the total daily energy intake in adults and children and constitutes 70% of the total added sugar in the diet [69].
